# Pathogenic Protist Transmembranome database (PPTdb): a web-based platform for searching and analysis of protist transmembrane proteins

**DOI:** 10.1186/s12859-019-2857-7

**Published:** 2019-07-24

**Authors:** Chi-Ching Lee, Po-Jung Huang, Yuan-Ming Yeh, Sin-You Chen, Cheng-Hsun Chiu, Wei-Hung Cheng, Petrus Tang

**Affiliations:** 1 Department and Graduate Institute of Computer Science and Information Engineering, Chang Gung University, Taoyuan, Taiwan; 2 Genomic Medicine Core Laboratory, Chang Gung Memorial Hospital, Linkou, Taiwan; 3 Department of Biomedical Sciences, Chang Gung University, Taoyuan, Taiwan; 4 Molecular Infectious Disease Research Center, Chang Gung Memorial Hospital, Linkou, Taiwan; 5 Department of Parasitology, College of Medicine, Chang Gung University, Taoyuan, Taiwan

## Abstract

**Background:**

Pathogenic protist membrane transporter proteins play important roles not only in exchanging molecules into and out of cells but also in acquiring nutrients and biosynthetic compounds from their hosts. Currently, there is no centralized protist membrane transporter database published, which makes system-wide comparisons and studies of host-pathogen membranomes difficult to achieve.

**Results:**

We analyzed over one million protein sequences from 139 protists with full or partial genome sequences. Putative transmembrane proteins were annotated by primary sequence alignments, conserved secondary structural elements, and functional domains. We have constructed the PPTdb (Pathogenic Protist Transmembranome database), a comprehensive membrane transporter protein portal for pathogenic protists and their human hosts. The PPTdb is a web-based database with a user-friendly searching and data querying interface, including hierarchical transporter classification (TC) numbers, protein sequences, functional annotations, conserved functional domains, batch sequence retrieving and downloads. The PPTdb also serves as an analytical platform to provide useful comparison/mining tools, including transmembrane ability evaluation, annotation of unknown proteins, informative visualization charts, and iterative functional mining of host-pathogen transporter proteins.

**Conclusions:**

The PPTdb collected putative protist transporter proteins and offers a user-friendly data retrieving interface. Moreover, a pairwise functional comparison ability can provide useful information for identifying functional uniqueness of each protist. Finally, the host and non-host protein similarity search can fulfill the needs of comprehensive studies of protists and their hosts. The PPTdb is freely accessible at http://pptdb.cgu.edu.tw.

**Electronic supplementary material:**

The online version of this article (10.1186/s12859-019-2857-7) contains supplementary material, which is available to authorized users.

## Background

Bioactive molecules that cross through the extracellular barrier mainly rely on channels, pores, or energy-consuming pumps composed of transporter proteins [1]. Transporters are specifically necessary for parasitic protists to salvage essential molecules for survival, such as nucleotides/ nucleosides, carbohydrates, and amino acids, from the human host [2–8]. In addition, membrane transporters also play a role in the drug resistance of protists [9]. These pivotal roles of transporters for parasitism strongly prompt parasitologists to characterize protist transporters in order to extend knowledge in protist pathogenesis and innovate new treatment strategies. Indeed, more than 50% of therapeutic drugs targeted to receptors or transporters on the membrane, emphasizing the importance of membrane proteins in disease control [10]. However, it is difficult to characterize the biological functions of membrane proteins because of uncertain experimental procedures [11]. Thus, a functional prediction based on protein sequence would be helpful to concentrate on the interested membrane proteins. Although whole genome sequencing of important human pathogenic protists has been conducted during the last decade, information about protist transporters has been lacking. Therefore, the goal of this study was to build the human Pathogenic Protist Transmembranome database (PPTdb) to provide classification and system-wide comparison to accelerate the exploration of protist transporters.

Several databases collecting transporter information have been constructed as a resource of known and putative transporter proteins. The Transporter Classification Database (TCDB) is the only classification organization for transporters that documents at least 1000 transporter families based on function and phylogeny derived from published studies [12]. TransportDB 2.0 has recorded potential transporters from ~ 2700 sequenced genomes using the following criteria: (1) primary amino acid sequence identity, and (2) predicted structural homology and topology [13]. PPTdb classification was based on protein sequence alignment to known human transporters, coupled with transmembrane domain prediction and gene ontology (GO) categorization to increase the accuracy of transporter annotation in sequenced protist genomes.

Advanced applications such as expression profiles and polymorphisms of transporters are included in the Human Transporter Database (HTD) [14]. The Yeast Transporter Protein database (YTPdb) contains membrane topology, post-translational modifications, and a “wiki-like” freely updatable platform [15]. The PPTdb is not only a resource but also an interactive functional search engine for potential protist transporters. The query section of PPTdb is composed of sequence identity to known human transporters and conserved transporter characteristics. The summary of a PPTdb search consists of a pairwise functional comparison of protist to human transporters that distinguishes either protist-specific transporters or human homologs. Furthermore, a functional comparison between two protists can be examined to highlight the commonalities between or uniqueness of transporters in each organism. An iterative search panel is another option for multiple functional queries in order to obtain more specific targets.

As the first transporter collection for protists, the PPTdb has globally analyzed more than one million protein transporters collected from sub-databases of the EupathDB family and from the NCBI genome archive. The classification of potential protist transporters depends on the sequence similarity to human host transporters, putative transmembrane domains, and GO functional groupings. The user-friendly interface allows one to use different strengths to filter out the desired transporters and to conduct inter-species comparisons to humans and other protists. Additionally, the iterative functional mining allows for the entry of more than one keyword to find possible transporters within one round of searching. Comparing to previous databases, PPTdb offers an easy-to-use data querying interface, such as human homolog gene filter, iterative functional mining, and PPTdb also contains regularly updated gene and amino acid data by a back-end automatically data processing pipeline. The PPTdb thus provides a platform for parasitologists to understand the field of transporters and discover new therapeutic targets in important protists by comparing human homologous genes and protist uniqueness transporter genes.

## Construction and content

### Sequence characterization and annotation

The data processing pipeline was mainly built by service-side command-line PHP and a suite of in-house developed text-processing and data retrieval modules which were implemented by Bash scripting language.

To gather the general and taxonomic information of each organism, we developed an automatic data retrieving and processing pipeline. Customized shell and PHP scripts were used to extract general and taxonomic information from NCBI taxonomy data portal. The taxonomy data were used to build the hierarchical species selection module on the front page of the website. To ensure that all the annotations were annotated using the same software environment, we re-annotated all the protein sequences based on functional and secondary structure information, such as transmembrane prediction by TMHMM, and functional annotations by PROSITE, Pfam, and Gene Ontology.

### Web-interface and database architecture

The PPTdb is a database providing real-time user interaction functionalities. Its user interface was implemented by HTML5, jQuery, and Ajax which can be opened by most modern web browsers. Functional annotations, species categories, sequence alignments and general information were stored in a relational database implemented by MySQL, which guarantees short latency and instant responses by online queries.

The PPTdb collected all protein sequence BLASTp results; traditional SQL queries such as ‘join’ and ‘nested-query’ require significant waiting time. To minimize the data querying time, we designed an SQL and PHP two-step conditional query methodology. The first step was called SQL query. Information for each species was separately stored in a MySQL data table, which can reduce SQL querying time, and multiple species queries could be executed in parallel. User-selected gene lists sent from the web interface were ignored in the first stage query and passed on to step 2, and all genes fitting the functional and sequence similarity conditions were selected from the database. The second step was the server-side gene query. The gene lists accepted from step 1 were stored in memory by creating a memory-cached search. According to our tests, this strategy could be executed at least twice as fast as a nested SQL query.

Amino acid sequences of all protist proteins are stored as FASTA files in our file-based database. A set of sequence-retrieving programs is used to extract user-selected gene sequences from web-based queries.

### Database contents

The PPTdb collected all known protist-annotated protein sequences from the EupathDB and NCBI genome archive [16–18]. There were 139 protist genomes composed of *Entamoeba* and *Acanthamoeba* (11 species), *Cryptosporidium* (12 species), *Giardia* (6 species), *Microsporidia* (26 species), *Piroplasma* (8 species), *Plasmodium* (23 species), *Toxoplasma* (26 species), *Trichomonas* (one species), and *Trypanosomatidae* (26 species). All the human transporter protein sequences were retrieved from the TCDB data portal which provides the comprehensive annotations of transporter sequences, including functional classifications. To construct human transporter sequences for pathogen and host studies, we selected all human transporter sequences based on the annotation records of TCDB. Table 1 shows the summary of database statistics.

**Table 1:** Database statistics of PPTdb

Category	Number of entries
Protists	139
Protein sequences	1,039,394
GO terms	1896
Transmembrane domains	465,391

**Fig. 1:**
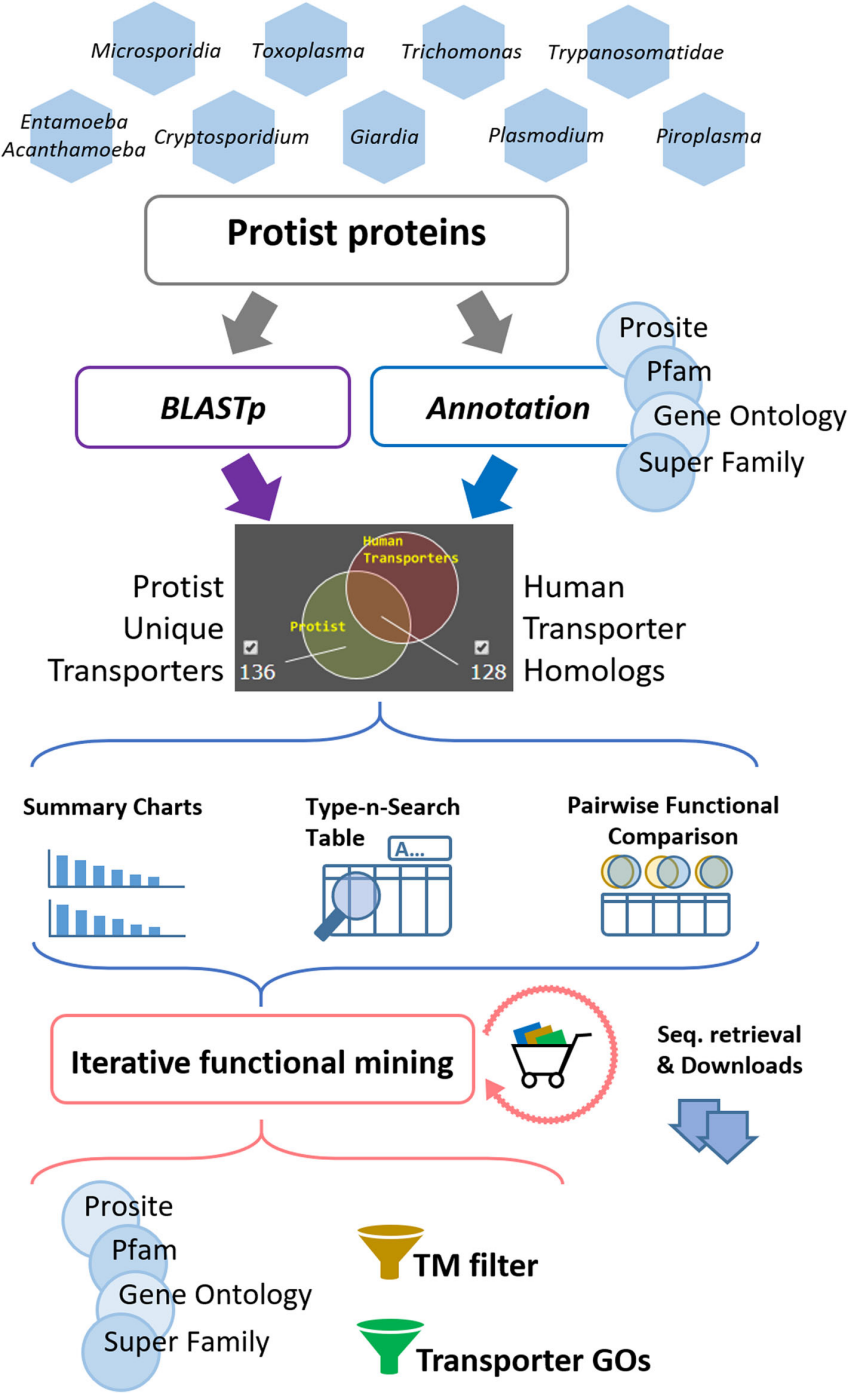
Analysis workflow of PPTdb Protein sequences collected were assigned functional annotations by the same software packages with equal parameters, including PROSITE, Pfam, Gene Ontology, and Super Family. The all-against-all primary sequence alignment executed by BLASTp was performed as well, and they were then stored in our back-end database. The system was armed with a high-speed database query system that allows the user to obtain the data instantly from the database. Four filters (transmembrane domain (TM), transporter GOs, iterative functional mining, and sequence homology) were used to help users to narrow their search results

The PPTdb serves as a knowledge portal, an online functional mining platform, and a cross-species comparison tool specific to protist transporter proteins and human hosts. It is freely accessible at http://pptdb.cgu.edu.tw. Figure 1 shows the analysis workflow and data mining processes of the PPTdb.

**Fig. 2:**
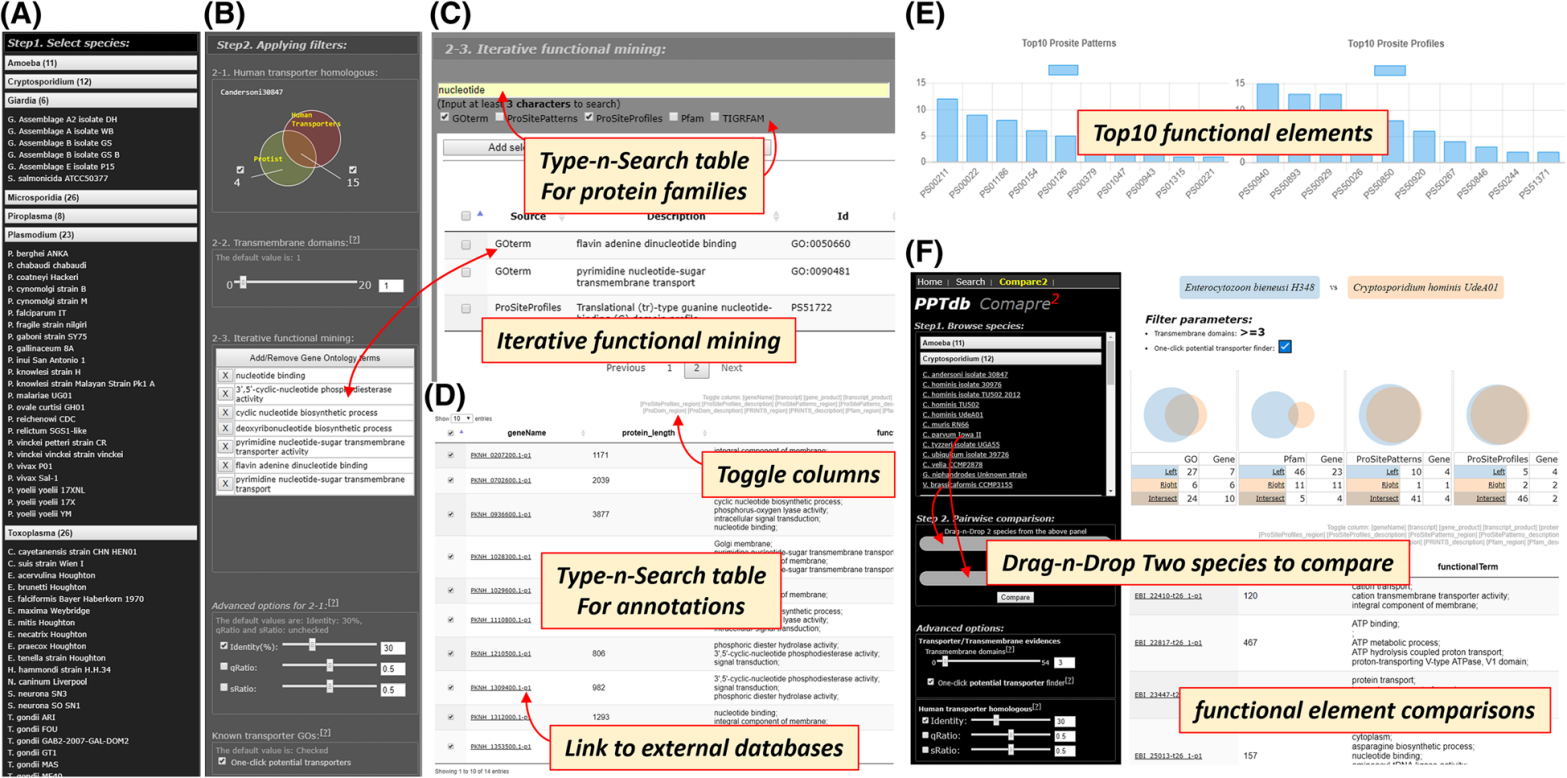
The interactive web interface (**a**) In step 1, users can choose interested species by clicking the species name of the species selector panel. The full genus, species and strain name is pop-up while the cursor hover on the species name. **b** Step 2 provides four kinds of filters. The filter 2–1 delivers the sequence identity results as a Venn diagram; the left (yellow circle) represents genes dissimilar to human proteins, while the intersecting region (color mixed of yellow and red) shows the human homolog transporter genes. Users can select either left, right, or both to narrow down the search gene sets. The filter 2–2 is the transmembrane-domain (TM) filter for the number of transmembrane domains allows users to select genes with a specific number of TMs. The iterative functional mining panel (filter 2–3), allowing the user to execute multiple-layer selection with multiple independent queries, shown in (**c**). All the selected functional description keywords of protein families including GO terms, Prosite patterns and profiles, Pfams, and TIGRFAMs are combined into a functional filter. The Advanced options contains the BLASPp parameter settings and known transporter GO filter. **d** The annotation table is implemented by type-n-search data table technology which allows the user to type any keyword characters, with or without complete spelling, to narrow down the search size. All the secondary search results can be passed into the middle panel, and the functional search can be performed iteratively. Users can be re-directed to the external databases by clicking the hyperlink on any underlined entries, such as GO IDs and gene IDs. Additionally, there is a check box on the first column of every entry in table which allows users to selected genes and apply to step 2 for further functional selection. **e** Genes filtered by four filters (TM filter, one-click transporter GO filter, sequences homologous filter, and iterative functional filter) are displayed by summary charts which list the top 10 dominant functional elements, including GO, PROSITE pattern, PROSITE profile, Pfam, and superfamily. The annotations, protein sequences, and BLASTp alignment results of selected genes are compressed into a single file and can be downloaded through the download link on the top side menu of the annotation Table. **f** Compare2 is designed for two protists systematically compared based on functional annotations. The Venn diagrams deliver the common or unique functional elements clearly, and all relative genes can be listed in the data table below

The database had collected 1,055,827 protein sequences which were annotated based on the same annotating software and parameters, including 465,391 transmembrane domains, 4429 Pfams, 1064 superfamilies, 1896 GO terms, 668 PROSITE patterns, and 695 PROSITE profiles. Figure 2 represents the interactive web interface of the PPTdb.

## Utility and discussion

### Transmembrane domain filter

The PPTdb annotated all proteins of all collected protists by TMHMM, which is the most widely used transmembrane domain (TM) prediction tool. Users could select transmembrane candidate proteins of interest with a specific number of TMs, such as a six-TM potassium channel, or a single TM domain for an alpha-helix. Using the same TMs could easily identify proteins with similar structures or biological functions, which was easier than searching an entire dataset.

### One-click potential transporter gene finder

We collected all 16,478 transporter proteins downloaded from the TCDB and annotated them by Gene Ontology terms to construct a transporter protein functional ontology dataset. Proteins collected in the TCDB share approximately 1300 GO terms (Additional file 1). Functional ontology terms were used to identify putative transporter proteins including functionally clarified and hypothetical proteins. Comparing to existing protist resources and collecting the feedbacks of testing members, the one-click potential transporter finder may be the most straightforward biological function-based gene finder for protists, of which annotations and sequences had not been comprehensively completed.

### Sequence homology search for human transporters

Primary sequence alignment is an effective method to identify proteins as human homologs. Protists and their human-host homolog genes could be used on studies of drug design and host-parasite interactions, because most functional elements share similar sequence identities, such as secondary structural domains and conserved functional elements. The PPTdb executed all-against-all amino acid sequence alignment by BLASTp on proteins of protist organisms and human transporter proteins adopted from TCDB. It also provided a sequence homology search interface allowing the user to preset the search criteria, including sequence identity, similarity, and a ratio of alignment length versus length of target or query proteins. A higher ratio indicates similarity to human proteins. The search result was visualized by a Venn diagram helping users to select or rule out human homology proteins. Users could click the checkbox on the Venn diagram to select protist-unique proteins, human transporter homology proteins, or both. Genes fitting the above criteria were summarized in functional component charts, and listed in Type-n-Search dynamic tables on the bottom side of the web page. Moreover, the table delivered transporter classification (TC) IDs for the human protein homologs which were identified by BLASTp alignment.

### Functional component charts

The selected putative transporter genes using the above filters were summarized using functional component charts by delivering the top 10 domains/components, allowing users to identify the most dominant functional elements from the genomic point of view. There were five functional component charts: GO, Pfam, PROSITE pattern, PROSITE profile, and superfamily. All of them were highly associated with biological functions.

### Dynamic type-n-search table

All the listed data including genes and GO terms were delivered by a Type-n-Search table, allowing users to narrow down the selection data. The data rows could be sorted by ascending or descending value (text data were sorted by alphabetical order, while the numeric data were sorted by number) by clicking the header in the data table. The keyword search box located in the top-right corner of the table could perform partial string matching for all columns in the table.

### Download page for sequence and annotation retrieval

The PPTdb provided a data retrieval tool for protein sequences and annotations targeting genes selected by the functional filters and homology search tool mentioned above. Download by annotations delivers the gene ID and annotations. Download by BLAST results contained all the primary sequence similarity results of users’ selected genes against all protein sequences recorded in the TransportDB. Download by sequences provided all amino acid sequences formatted in a FASTA text file. All three download links were dynamically generated by the user modifying any search parameters above. Files were zipped in a tar.gz format which could be unzipped by conventional file compression tools such as 7-zip in Windows and MacOS or tar in Linux operating systems.

### Iterative functional mining

Most protist resources provided basic search functionalities including a gene ID/name or keyword search. However, if a user wanted to search by specific biological function such as “calcium channel”, it was not easy to ensure that everyone can type the correct term. The PPTdb provided a real-time GO description search. Users could type only a few characters of the description, and the system searched our back-end database in the background and returned suggested terms by the Type-n-Search data table. In contrast to other auto-filled search boxes (such as Google search), the user could only select one suggested option. In our iterative functional mining interface, users could do the secondary search using the query box in the top-right corner of the data table to narrow down the result from hundreds of returned elements. All the suggested elements could be added into a collecting box which was similar to a shopping cart in the online shopping website.

Unlike other search interfaces that only allowed the user to use single terms for database searching, the collecting box supports multiple iterations of searches. Items returned from the database of every search could be put into the collecting box together (Fig. 2c). Moreover, items put into the collecting box could also be removed by clicking the “x” button on the left-hand side of each item (Fig. 2b). For example, users could use “calcium channel”, “ion channel”, and “voltage-gated channel” as keywords to query the database in three independent searches and add all the items into the collecting box. There were 96 GO descriptions associated with “ion channel”; then, the secondary search could be used to remove all 9 “ligand-gated” items in the search results. Finally, all 90 items associated with “ion channel” except 9 “ligand-gated” items could be added into the collecting box. The collecting box serves as a functional filter that could be coupled with the previously mentioned TM filter, the one-click potential transporter finder, and the human transporter homolog filter (Fig. 2b). These four filters comprise the “iterative evidence-driven putative transporter system mining” workflow of PPTdb.

### Pairwise functional compositional comparisons

Interspecies comparisons of current protist resources focused on the number of genes, transporter classes, or sequence similarities. However, constraint-based comparison, which allowed users to pre-define specific search parameters or select a subset from the whole genome, was still lacking in protist resources. The PPTdb provided pairwise functional element comparison through the Venn diagrams. Users could select unique (left- and right-hand side) or shared (intersecting region of the Venn diagram) functional elements. After that, the Type-n-Search data table delivered genes that contain selected functional elements for users’ further detailed investigations.

### Data retrieving and automatically update functionality

There were several automatically updating scripts to guarantee the latest’s data of PPTdb, including data retrieving, gene annotation, data processing and database renewwing. PPTdb were consisted by two exactly the same virtual machines – the stable version for public use and the standby version for latest data integration. Once all the data was completely updated and deposited into PPTdb’s database, the standby version would go on-line. And the stable version will go off-line for next data updating.

### Comparison to other protist transporter resources

Currently, there was no published transporter-system database specifically designed for protists. Two general-purpose transporter protein databases (TCDB and TransportDB) could be used for protist transporter system studies. However, both databases collected a limited number of protists, which could not provide sufficient resources for protist transporter protein studies. The TCDB contained data for more than ten thousand transporter proteins and provided a transporter classification (TC) system recognized by the International Union of Biochemistry and Molecular Biology (IUBMB), generating systematical functional categories. However, there were only 75 protists in this database and fewer than 300 protist transporters. TransportDB provided clean and comprehensive data resources for transporter systems targeting to sequenced genomes which contained ~ 2500 bacteria species; however, there were fewer than 40 eukaryotic species (and only 11 protists) in the latest published version.

Both TCDB and TransportDB provided interfaces for transporter protein studies; however, neither of them offered mining tools for protist versus human homolog gene searches, protist to protist comparison, or further protist-associated studies. Table 2 listed the major differences between PPTdb, TCDB, and TransportDB from the protist research point of view.

**Table 2:** A comparison table between PPTdb and other protist transporter resources

Tool	PPTdb	TCDB	TransportDB
Protist Genomes	139	75^a^	11
Designed for	Protist-human transporter studies	Transporter classification	Transporter knowledgebase
Annotations	■	■	■
Human homology search	■	■	–
Functional keyword search^b^	■	□	□
Pairwise protist comparison	■	–	–
Visualized comparison results	■	–	■
Integrated Transport classification id	■	■	–

■: full function; □: partial function; −: NA

^a^cross reference to EupathDB by species name

^b^PPTdb provides iterative functional mining, other resources offer only one-way search

### Iterative functional mining workflow: an example of use

Horizontal gene transfer events were known as evolutionary driving forces of eukaryotes [19]. For example, nucleotide transporter (NTT) gene acquisition was reported as a major evolutionary innovation of *Microsporidia* which were intracellular parasites of animals and human [20].

As a proof of our user-friendly interface, sequences of putative NTTs were identified from seven *Microsporidia* species using PPTdb’s iterative functional mining workflow. By using traditional one-way search interface that only allowed single query once a time, users must enter an exactly correct term, for example, “nucleotide transport” returned only 1 GO descriptions in *A. Algerae PRA339*. This was because words between “nucleotide” and “transport” could not be searched by the general query method. In iterative functional mining, one could first search word “nucleotide” which returned all GO descriptions which contained keyword “nucleotide”. Then, the type-n-search data table of PPTdb allowed a secondary refining search by entering “transport” to narrow down search results. This two-way search interface offered a high flexibility especially on multiple keyword combinations which could not be searched by one-way search, for example, nucleotide-sugar transmembrane transporter activity (GO:0005338), guanine nucleotide transmembrane transporter activity (GO:0001409), and nucleotide transmembrane transporter activity (GO:0015215). Moreover, the type-n-search table instantly returned database query candidates. Users could obtain putative results by just entering a few characters instead of entire searching keywords. For example, entering “transport” would return all the candidate entries including “transport”, “transporter”, and “transmembrane transporter” which could be act as a search guidance to inform users that there was more than one “transport” associated keywords in the GO description database.

In this example, 22 putative transporters were identified by those nucleotide transport associated GO descriptions from seven *Microsporidia* species (Table 3). Interestingly, more than half of the results are hypothetical proteins. The protist uniqueness genes and human transporter homologs could be easily separated from the search. Finally, we could download all the FASTA sequences by clicking the Download link of the web page. Tools such as MAFFT [21] and ClustalW [22] could do the multiple sequence alignment and deliver the phylogenetic tree of these sequences. Detailed information of steps would be found on the demonstration page of PPTdb (http://pptdb.cgu.edu.tw/demo.php).

**Table 3:** Search entries^a^ of NTTs in seven Microsoporidia species

Species	Homology search	NTT (putative transporters)	Transcript Id	Annotation	Protein Length
*A. algerae PRA109*	Portist uniqueness	2 (60)	H311_01875	hypothetical protein	250
			H311_05190	hypothetical protein	120
	Human homolog	2 (59)	H311_02925	hypothetical protein	562
			H311_03867	hypothetical protein	327
*A. algerae PRA339*	Portist uniqueness	1 (51)	H312_02827	hypothetical protein	539
	Human homolog	1 (49)	H312_02469	hypothetical protein	562
			AEWD_081300	ATP/ADP translocase	559
			AEWD_100320	ATP/ADP translocase	536
			AEWD_100430	ATP/ADP translocase	543
*E. cuniculi EC1*	Portist uniqueness	4 (41)	AEWD_100450	ATP/ADP translocase	553
	Human homolog	0 (41)	–	–	–
*N. bombycis CQ1*	Portist uniqueness	1 (80)	NBO_251gi001	ADP/ATP carrier protein 1	206
	Human homolog	2 (59)	NBO_55g0018	ADP,ATP carrier protein 1	484
			NBO_100gi003	ADP/ATP carrier protein 1	151
*P. neurophilia strain MK1*	Portist uniqueness	2 (37)	M153_7540001844	ATP:ADP Antiporter (AAA) Family	568
			M153_1368000483	ATP:ADP Antiporter (AAA) Family	568
	Human homolog	1 (57)	M153_32600001719	ATP:ADP Antiporter (AAA) Family	560
*V. corneae ATCC 50505*	Portist uniqueness	3 (56)	VICG_00013	hypothetical protein	240
			VICG_00014	hypothetical protein	329
			VICG_00919	hypothetical protein	609
	Human homolog	0 (46)	–	–	–
			VCUG_00366	hypothetical protein	530
*V. culicis subsp. floridensis*	Portist uniqueness	2 (57)	VCUG_00419	hypothetical protein	552
	Human homolog	1 (44)	VCUG_01129	hypothetical protein	566

^a^The parameters used in this example: BLASTp identity: 30%; one-click-transporter filter: on; Number of transmembrane domains ≧ 1

### Specific search strategies for putative transporter proteins

PPTdb offered the precisely functional search instead of general keyword search used by general purposed databases such as Entrez Gene [17, 18], UniProt [23], and EuPathDB [16]. In addition to keyword search, PPTdb also provided specialized pre-set search filters for putative transporter proteins, including one-click-potential transporter genes, number of transmembrane domains, and iterative functional search boxes. Table 4 illustrated a search term “nucleotide” for putative transporter proteins with 1 transmembrane domain of species *Acanthamoeba castellanii* str. Neff. It was complicated to mimic all possible search movements for every user of these databases that provided several advanced search tools. The most straightforward search strategy was the keyword search and filters provided by each database. The search would be set if the database offers pre-set filters, such as number of transmembrane domains. However, the keyword search was used without pre-set filters, such as putative transporter proteins. The result showed that the functional filter and iterative search functionalities could make the search more user-friendly than other general purposed databases. All the searched genes Ids from PPTdb could be downloaded in a text format and then be executed more detailed data retrieving processes, such as genetic information from Entrez Gene database, protein 3D structures and pathways from UniProt database.

**Table 4:** Search comparisons PPTdb and several general purpose genomic databases

Items	PPTdb	Entrez Gene	Uniprot	EuPathDB
*Search term*	nucleotide	nucleotide AND transporter AND *Acanthamoeba castellanii* str. Neff.	nucleotide transporter *Acanthamoeba castellanii* str. Neff.	nucleotide
^*a*^ *Pre-search settings*	Click *A. castellanii* str. Neff			
	Set number of transmembrane domain: 1	–	–	Click AmoebaDB
	One-click potential transporters: checked			1135 genes for keyword search
*Number of searched items*	12 GO terms			76 genes with 1 transmembrane domain
	3 Prosite Patterns	No number of transmembrane domain filter	No number of transmembrane domain filter	
	5 Pfams			^c^0 genes for annotation with “transporter” as keyword
*Number of Genes after searching*	105	^b^303	^b^290	^c^0/76

^a^There are several search combinations of each database, it is reasonable that different search combinations may lead to different search results. This demo demonstrates one search strategy of each database

^b^No transmembrane domain filter

^c^There is no specific column for putative transporter genes of EuPathDB. Using “transporter” as keyword is the most possible search action used by most users

### Future work

PPTdb collected all the putative transporter protist proteins and provides a user-friendly data querying interface. The next goal is to collect the human validated transporter proteins by offering a system for putative and validated proteins comparison. The collection of 3D structures of protist transporters will be the goal as well. We believe these future works will make PPTdb more useful on potential protist associated treatment or drug development.

## Conclusions

The PPTdb had been specifically designed for protist transporter system studies and provides a data query portal, an online comparison tool, and a flexible functional search interface. For all putative, hypothetical, or curated protist proteins, the PPTdb provided functional annotations. The PPTdb also offered a straightforward protist-human homology search interface for pathogen and host studies.

## Additional file

Additional file 1: GO terms used in the PPTdb (XLSX 45 kb)

## Abbreviations

BLASTp: Basic local alignment search tool for protein
GO: Gene Ontology
HTD: Human Transporter Database
HTML5: HyperText markup language 5
IUBMB: International Union of Biochemistry and Molecular Biology
MySQL: My structured query language
NCBI: National Center for Biotechnology Information
NTT: Nucleotide transporter
PHP: PHP hypertext preprocessor
PPTdb: Pathogenic Protist Transmembranome database
TC: Transporter classification
TCDB: Transporter classification database
TM: Transmembrane domain
TMHMM: Transmembrane Helices; Hidden Markov Model
YTPdb: Yeast Transporter Protein Database
